# “Let us define ourselves”: forced migrants’ use of multiple identities as a tactic for social navigation

**DOI:** 10.1186/s40359-021-00630-6

**Published:** 2021-08-25

**Authors:** Dieu Hack-Polay, Ali B. Mahmoud, Maria Kordowicz, Roda Madziva, Charles Kivunja

**Affiliations:** 1 Crandall University, Moncton, Canada; 2 University of Lincoln, Lincoln, UK; 3 St. John’s University, New York City, USA; 4 University of Wales Trinity St David, Lampeter, UK; 5 University of Nottingham, Nottingham, UK; 6 University of New England, Armidale, Australia

**Keywords:** Displacement, Migrant, Social navigation, Situatedness, Social transactional perspective

## Abstract

**Background:**

The article examines how and why multiple identities are altered, used and discarded by forced migrants.

**Methods:**

The research is located in the constructivist paradigm. We used thematic analysis to analyse data gathered through interviews with nineteen forced migrants.

**Results:**

We found that, though individual migrants can make deliberate choices about which identities to be associated with, they are constrained in the process by external socio-economic factors that lead them to adopt identities that are perceived to be advantageous to navigate the new social system. Moreover, the construction of forced migrants’ identity includes significant contextuality, transactionality and situatedness.

**Conclusions:**

Our research contributes to the literature on migrant identity practice concerning the stigma associated with forced migrant status and the extent to which migrants appraise their reception in exile as undignified. Additionally, examining migrant identities allows the researchers to apprehend the diverse facets of identity as far as migrants are concerned. Future research may draw a larger sample to examine other impactful dimensions of identity fluctuation, e.g. gender, education, social media, the extent of prior trauma, etc.

## Background

Recent data by the United Nations High Commissioner for Refugees (UNHCR) shows that nearly 80 million individuals worldwide have been forced to leave their homes, leading to nearly 26 million refugees, of whom, around half, aged younger than 18 years old [1]. For forced migrants, countries of origin are places of violence, cruel wars, and conflicts. They can face hazardous dangers and threats to one’s or their family members’ lives embodied by political persecution, religious intolerance, and pressure to join militias, sexual violence, etc. [2]. Earlier scholarship into migrant identity negotiation has examined the subject drawing on the experiences of people from diverse cultures, e.g. the Somali community, Syrians, unaccompanied minors [3–7]. However, the key strength of our work is that it draws attention to the link between migration and race, given the centrality of race in driving British public attitudes towards immigrant groups. Migration scholars [8] have drawn attention to the ways in which race and racism relate to migration. Focusing on the UK context, these authors have conceptualised ‘race as a political project rooted in colonialism and imperialism,’ and how race is increasingly used to categorize immigrants, especially those from the global south as the ‘other’; hence different and inferior [8]. U Erel, K Murji and Z Nahaboo [8] go further to show how the race-migration nexus makes visible multiple and co-existing stratifications that emerge through racialization instead of a singular in-group/out-group continuum upon which all migrants (and settled communities) are mapped’. Indeed, race and migration are connected in complex ways, and this has a significant impact on how different groups of migrants realise full integration into British society. Existing research [e.g., [9, 10] shows that public attitudes towards immigrants in Britain (as in Europe) are framed along racial and ‘ethnic hierarchies’ with the most preferred groups being those who are white, English-speaking and from Christian countries and the least preferred being those who are non-white, Black and Muslims. Particularly with regards to Black immigrants, the Fundamental Rights Agency, in their 2019 report, observed that across Europe black Africans increasingly face widespread and entrenched prejudice, exclusion and hurdles to inclusion and integration are multi-faceted [11].

Therefore, exploring individual perceptions of identity would contribute to a greater understanding of forced migrants’ lived experiences. In this article, we use the term forced migrants to encapsulate migrants that were coerced into leaving their country of origin, e.g. persecution, political unrest or environmental upheavals [12]. This study examines how forced migrants negotiate and articulate multiple identities, including the strategic deployment and concealment of ethnic and refugee identity, as they navigate different political, emotional and social spaces. The overarching research question was: How do individual perceptions of identity affect the lived experiences of forced migrants?

The continuing refugee crisis in Europe [13] and the flow of forced migrants continuing to attempt to cross into Europe and the United States suggests that forced migration remains a burning issue. This has fuelled the growth of research on the psychological and social processes experienced by migrants themselves [14]. It further raises questions around identity shifts as forced migrants attempt to navigate new [and often harrowing] experiences and societies. It is well chronicled that cultural distance can lead others to misinterpret and misunderstand migrants’ motives for embracing, rejecting or juggling new identities [15, 16]. For instance, previous research shows that when individuals of an ethnic minority or sub-group prefer a hyphenated cultural identity, it can sometimes be crucial for the dominant ethnic group to acknowledge this identity during intergroup interactions [2]. Since biculturalism ought to be a cultural asset for cohesive societies [17], it is, therefore, vital that ethnic minorities’ desired identities are accurately understood and recognised [2], especially amidst the challenges facing the integration of migrants in the host countries [18]. Our work investigates the extent to which some forced migrants use identity for gain, for instance, to receive psychological and social benefits from articulating different socio-cultural and national identities in the host country. In doing, we aim to create greater insight into the relationship between identity and social navigation.

The paper is organised as follows: The first section of our paper examines the critical literature surrounding migrants and identity. We then detail the methodological framework used to conduct this research. We then present the results with commentaries before a critical discussion of our findings and study strengths and limitations. Finally, we draw conclusions that summarise the key perspectives and identify future research possibilities.

## Literature review and theoretical foundations

G Valentine and D Sporton [19] argue that ‘the twin forces of the global economy and global conflicts’ have accelerated and transformed international migration patterns in the twenty-first century, raising questions about how such mobility might shape processes of identification and/or identity formation. R Jenkins [20] notes that identity, as ‘our understanding of who we are and of whom other people are,’ has come to be something that is ‘managed’. The argument is that identity—in the age of migration—is not an inherited, ascribed, nor achieved status that matters, but the status that one ‘maintains’ in any given place and time in the process of fitting oneself into a community of ‘strangers’ [21, 22].

A Giddens [23] coins the phrase ‘identity project’, noting that in late modernity, the self ‘has to be reflexively made’ in order to be able to respond to the anxieties raised by rapid social change. Here the argument is that self-identity can no longer be taken to be ‘something that is just given’ but has to be understood to be ‘something that has to be routinely created and sustained in the reflexive activities of the individual’ [23]. The main reason for this, as A Giddens argues, is that modern societies no longer offer stable ‘anchor points’ for the self, consequently leading to the construction and reconstruction of the self as a response to and way of coping with the uncertainties [23]. This means that, for an individual to maintain regular interaction with others in the day-to-day world, they must constantly integrate events that happen in the external world and classify them into the ongoing *story* about the self’. For this paper’s purpose, we define identity as the story about the self [24]. Further, the notion of how life events can shape people’s identities in the era of globalisation has gained much currency in the migration field, where identity construction is seen as involving lived experiences as well as a mental state susceptible to sustain significant life changes that involve place, people, culture, and so forth [25, 26].

Meanwhile, it has been noted that migrants are significantly more exposed to identity change than other social groups [25, 27, 28]. The flight of forced migrants into exile results in a loss of identity. Moreover, their arrival is often marked by uncertainties that raise questions about belonging and identity. This often leaves mental scars and sometimes physical footprints, which alter the way their life course evolves and even the manner in which they talk about themselves. Giddens argues that ‘What to do?’ ‘How to act?’ and ‘Who to be?’ are questions affecting everyone in modern societies, prompting an identity crisis in each of us [23]. Therefore, if we view forced migration as inextricably associated with modernity—as other authors [25, 29] tend to accept—then Giddens’ point is relevant to analysing the identity issues regarding forced migrants [23]. However, for forced migrants, the magnitude of identity crisis is more pronounced given the spatial, demography, temporal, economic and cultural dislocation.

Thus, as SS Kebede [26] argues, in the context of forced migration, forming and reforming identities are part of the struggle to ascertain belongingness to a new socio-cultural domain. This assertion entails occasionally dramatic deconstruction and reconstruction of self and its association with various communities and identities [26, 30, 31]. In examining the process of deconstruction of forced migrants’ identity, JW Berry’s concept of mutuality in acculturation is helpful [32]. JW Berry [32] argues that mutual contacts and interactions affect migrant and host groups’ acculturative choices and desired outcomes. J Arends-Tóth and FJRVD Vijver [33] examined mutuality in acculturation in the Dutch context. They found mutual agreement (at least in the public sphere) between native Dutch and Turkish minorities about the need for minorities to integrate [33].

However, in the private domain, migrant minorities expressed a preference for identity pluralism. This shows that that identity construction can be domain-specific and contextual [33, 34]. MM Doucerain [34] particularly identifies dynamics within the individual, home country and the wider new social environment as the key contextual factors that influence acculturation and identity formation. The process can be painful and disconcerting since deconstructing the self implies dismantling deeply rooted assumptions that make the self and define its relationships with the group. MK Kumsa [31] sees this process as shifting spaces of belongingness. Reconstructing oneself may entail cultural and societal aspects that may not neatly fit the ‘old engine’ (the old self), causing a struggle to fit and sometimes ‘halfway’ working of the new parts. From JD Pugh and MK Kumsa perspectives [25, 31], this is about negotiating identity, an essential pre-requisite to the (re)definition of self and to belonging. H Zagefka and R Brown found that immigrants who displayed a relative fit had a greater chance of acceptance into German society, reinforcing the view that belonging derives from immigrants’ appraisal of the host society's expectation and developing ‘acceptable’ identities [35].

At the same time, it has been long established that forced migrants increasingly embody multiple and fluid identities in different spaces and times. For example, in their exploration of Somali refugees’ experiences in the UK, G Valentine and D Sporton show that identity construction or formation does not necessarily occur in a vacuum but is relational in nature, being attained through interaction with others and in and through different spaces [19]. Thus, they observe that ‘one identity category may be used to differentiate another in specific spatial contexts, and particular subject positions may become salient or irrelevant in particular spaces’ [19]—see also MM Doucerain [34], J Arends-Tóth and FJRVD Vijver [33] and M Navas, MC García, J Sánchez, AJ Rojas, P Pumares and JS Fernández [36]. However, the major challenge with regards to embodying multiple and shifting identities, as G Valentine and D Sporton [19] argue, lies in that a given identity is not just something that an individual can claim; instead, it is also dependent, at least to some extent, on an individual’s identity being accepted or recognised by others.

Such assertions are supported by R Madziva [37], who in her research with Christian asylum seekers from the Muslim majority countries notes that, although Pakistani Christians increasingly emphasised their Christian identity above their ethnonational identity in their narratives of the self, immigration officials, on the contrary, took Pakistani as a proxy for Islam. In this context, as R Madziva argues, visible identity (bodies) played a ‘significant role in blurring religious boundaries and nullifying the distinctiveness of the participants’ Christian identity’ [37].

This resonates well with the arguments of JD Pugh [25], T Polzer Ngwato [38] and M Navas, MC García, J Sánchez, AJ Rojas, P Pumares and JS Fernández [36] that identity formation or reformation in exile entails efforts to render certain identity characteristics visible or invisible (and to some extent audible and inaudible) depending on place but also as influenced by the identities of people the forced migrants encounter or enter into personal conversations with. However, earlier scholarship into migrant identity negotiation has examined the subjective experiences of migrants [3, 27, 39] but has mainly focused on group experience, for instance, the Somali community, Syrians, unaccompanied minors, and others. While some research has also considered individual experiences [4], we argue that more research is needed to increase our understandings of how individuals navigate forced exile from an identity perspective.

In this article, we endeavour to explore the experiences of forced migrants from diverse backgrounds and of different nationalities that the first author interviewed in the UK. In the same perspective as AB Kuyini and C Kivunja [40] and G Valentine and D Sporton [19], we focus on the ‘multiple, shifting and sometimes contradictory ways in which individuals identify and disidentify with other groups’ and with their fluctuating emotional investment in different subject positions. In so doing, we seek to show how our participants worked hard to try to minimise the signs of difference that set them apart as the ‘other’ as a strategy to reduce social distances between themselves and the host population. This indicates the subjectivities attached to the notion and expression of identity, which is formed by the social actors through their stories and lived experiences [41]. This means that the contingencies of a given time and space can lead a social actor to willingly espouse a variety of selves [42, 43].

## Methods

As this research set out to understand the identities forced migrants construct from their lived experiences, we located it in the interpretivist methodological paradigm. This was an appropriate paradigm for our work because it espouses the assumption of a subjectivist epistemology. As M Crotty [44] explains, this epistemology holds that the real world does not exist separately from our understanding of it. Instead, we know what we know because of our interactions and experiences with real-world phenomena [44].

Another reason why the interpretivist paradigm was chosen for this research is that it also assumes a relativist ontology. As EG Guba and YS Lincoln [45] explain, this ontological theory of interpretivism is relativism. Relativism is the belief that reality is subjective and differs from person to person [45]. The third reason we located this research within the interpretivist paradigm was that its methodology assumes experiential-naturalistic-inductive processes in gathering, analysing, and interpreting data.

The fourth reason for choosing this paradigm is its assumption of value-laden axiology. This assumption holds that whatever knowledge we gain through research is value-laden because researchers assert their values and beliefs when they choose what to research, how to conduct the research, and how to interpret the data [46], as was indeed the case in deciding our research design and data analysis strategies as outlined below.

We designed this empirical research to use the thematic analysis approach. Yin’s explanation informed our choice of this method—that an empirical inquiry examines a contemporary phenomenon thoroughly and within its real-world context [47]. The thematic analysis allows the researcher to grasp the participants’ narratives and extract vital meanings from their real-life experiences as relayed in their own words [48]. This objective of gaining a deep understanding of the subject was indeed the impetus for the present study, which sought to learn about the identities that forced migrants to construct in their new countries. The method was most suitable for this research because thematic analysis helps ‘interrogate the various meanings that subjects attach to phenomena’ [48, 49]. As pointed out by AB Kuyini and C Kivunja, ‘moving to another country is associated with loss at different levels, and issues of identity, power/influence and knowledge habitus are all at play [40]. These issues tend to be exacerbated when such migrations are forced, as in the case of the forced migrants interviewed in this research. Thus, this analysis method was fit for our research because we set out to understand migrants’ perceptions of their identities in their new countries.

In our research, we decided to include only forced migrants into the UK who had resided in the UK for a minimum of 3 years. We envisaged 3 years as a reasonable time for the immigrants to have enough experience about identity issues in their new country. Participant selection applied a convenience-sampling, snowballing strategy. The snowball approach was unlikely to have a confounding impact on the data by the fact that it was difficult for the researchers to know which forced migrant has been involved in the use of multiple identities. Thus, the initial participants contacted became aids for the researchers to identify and filter suitable participants who closely met our selection criteria. Following this strategy, one of the authors approached an acquaintance, a former work colleague who introduced the first participant (female) who, through her network, led the researcher to other respondents meeting the three-year UK residence criterion. Applying this strategy, we interviewed 19 forced migrants, who provided narrative data. However, the participants were unrelated. It was suggested to the first contact that the researchers wanted someone who was not related to them (e.g. husband or sibling) and preferably from a different country. This was to minimise bias and to enable us to collect a variety of experiences. As indicated earlier, the migrants interviewed had been in the United Kingdom for over 3 years at the time of the semi-structured interviews. The interviews were conducted in early 2018 in London. They lasted one hour on average and were recorded manually. Most participants were millennial (79%) men (58%), and all came from Sub-Saharan African countries. The participants’ details are shown in Table 1 below:

**Table 1:** Participant details

Participant	Gender	Age	Country of origin
P10	F	29	Congo (DR)
P15	F	25	Congo (DR)
P9	M	23	Congo (DR)
P6	M	35	Congo (DR)
P4	M	27	Ivory Coast
P16	M	29	Ivory Coast
P17	F	32	Ivory Coast
P3	F	37	Sierra Leone
P5	F	40	Sierra Leone
P18	F	36	Sierra Leone
P8	M	39	Rwanda
P19	F	43	Rwanda
P1	M	35	Sudan
P11	M	28	Sudan
P7	M	31	Somalia
P12	F	36	Burkina Faso
P13	M	26	Burkina Faso
P14	M	33	Senegal
P2	M	42	Gambia

The interview questions were framed in a way that the participants could provide independent answers not alluded to by the interview question. For example:’Have you ever pretended to be someone you are not?’. The questions used common terms that led the participants to tell their stories themselves.

All interviews were recorded and transcribed, and separately and then collectively analysed by the researchers thematically. The analysis started with transcript re-reading for data familiarisation. We then engaged in open coding so that individual transcripts were systematically reviewed through a process of iteration to derive the themes and meaning emerging from the contents. To improve the rigour of the procedures employed in this study [50], we offered all the participants the chance to review the interview transcripts and revise them (if needed). Out of all of the participants, only half chose to revisit the interview transcripts. The data analysed through open coding, as the researchers reviewed the transcripts to ascertain the meaning of the participants’ narratives. We aimed to make sure that we had an in-depth understanding of how participant tell their stories, namely, what identities, behaviours, activities, events, relationships and shared meanings are conceived through language [51]. A coding structure was developed, which captured the distribution of narratives of the participants and aspects of perspectivization [52]. We colour-coded the data to capture similar ideas to produce five broad categories: identity denial, identity borrowing, identities as situated choices, identity as a social integration strategy, and identity as psychological healing. It helped us identify the converging and diverging themes through iterative discussion that were then refined to arrive at two final analytical themes: Constructing new identities as psychological healing and identity fluctuation as a social navigation tactic.

Confidentiality and anonymity were critical to address since participants had personal stories. The disclosure of which to others could affect their confidence as social players in the community or willingness to participate in future research in the field (see [37]). The interviewees made statements connected with previous life and religious practices, which contrasted with norms in the new communities, and these aspects required researcher sensitivity to protect the subjects’ identities and privacy. Thus, the researchers have protected participants’ identities by using pseudonyms to present data and discuss the findings. Informed consents to take part in the study were obtained from the participants. Each participant was asked for his/her personal consent to participate in the research and was given assurance. They were reassured that if they did not participate, there would be no negative consequences. Furthermore, each participant was asked for consent to let us tape-record the interview. The study was approved by Crandall University’s Research Ethics Committee. All methods were performed in accordance with the relevant guidelines and regulations.

## Findings

The analytical process enabled the researchers to capture the meanings and perspectives framed around interpretative repertoires, ideological dilemmas and subject positions. We present the participants’ key statements to document the frames of analysis.

### Constructing new identities as psychological healing

The issue of human displacement in the African context is clearly captured in the volume edited by M Utas [53] “African Conflicts and Informal Power”, which presents case studies from a variety of African countries, settings and institutions, and showing armed conflicts and wars as the common factors that displace people. To a large degree, all the migrants we interviewed fled violence and persecution in the country of origin. They saw their arrival in the United Kingdom as a flight to a safe haven. The migrants’ narratives support this assertion well. Accounts provided by P1, P2 and P3—support this feeling that was prevalent in the migrants’ narratives:

Leaving beautiful Sudan was extremely painful. The military conflicts drove us out. We had to find a safe place to be. I didn’t stay in the first African country I reached (South Africa) because foreigners were not welcome there— (P1).

I went through three different countries before landing here in UK. When I fled The Sierra Leone due to civil war; Cameroon is where I went for safety. But Cameroon also started to have civil wars; so I came to UK through Libya (P2).

My uncle was shot and died alongside his three children. Only his wife survived but then it was terrible. She was sexually assaulted several times by soldiers. Seeing what the rebel soldiers were doing in town, I used all monies I had to pay those who could help me leave because I fear the same fate— (P3).

Having escaped from war-torn and life-threatening environments, their arrival in the UK marked the beginning of a new ‘social navigation’ process. As argued by H Vigh [54], the concept of social navigation makes it possible to focus on how individuals move within changing social environments. Here we seek to show these migrants’ expressed efforts to survive and forge a future for themselves in a new and increasingly changing environment, characterised by racism and discrimination. For their first steps in the new community, the migrants did deliberately change their identities from time to time to have their dignity protected by other selves that they saw as positive identities. However, most participants showed unease about the pity that locals appeared to exhibit towards them. In some instances, the migrants received less favourable treatment and other times more favourable treatment than the average person; in most cases, the migrants resented the ‘patronising’ [in P6’s words] aspects of the way they were dealt with. Two of the participants’ narratives translate well this sense of belittling of the migrants in the host country:

As long as I claim to be and mimic British accent, straight away people are kinder. Then they do not consider as foreign as much. You can then have some meaningful conversation with them — (P4).

When women see you as a foreigner, automatically they think you want to be with them for immigration reason. When you say you are British, they trust you more because they believe in your genuine love — (P5)

When people in my church knew I was a refugee, there was a huge sense of pity. Some people offered me clothes and even small amounts of money. I felt uncomfortable. Some others kept their distance. I hate being the constant focus of attention. I prefer that people don’t know my refugee background. That’s better and I live with more dignity and pride — (P5).

In their study with African asylum-seeking women, M Clare, S Goodman, H Liebling and H Laing [55] note how ‘participants used two interacting repertoires, ‘rejecting pity’ and ‘being strong’, to resist inferior positions. Thus, they argue that ‘by constructing themselves as strong and not needing pity, participants positioned themselves as in control of their lives’.

In our study, the migrants’ narratives conveyed their sense of discomfort because they felt that they were viewed only as immigrants instead of full members of the new communities. These feelings were widely shared and were well mirrored in the narratives of P6, P7 and P8.

### Situated and contextual migrant identities

Our participants were individuals who inhabited a social space of ‘restricted possibilities’ [7]. Their navigation of the social space reveals their struggles to escape confining structures and circumstances as they moved under the influence of multiple forces. Thus, when asked to state whom they thought they were, the participants pointed to context-related identities. They showed awareness of both the multiple forces restricting them and the identities carried. However, they were also conscious of the situatedness of these selves, meaning that the identities were expressed differently depending on time, place and social entourage, and so forth. For instance, the migrants would claim certain national or linguistic identities in a social setting and other identities in different milieus. These constant fluctuations were opportunity-driven, both psychologically and materially, for example, to command respect and dignity or find employment or better housing. The following participants’ narratives exemplify this situation:

Really, I try to tailor my person to various environments. If I stay the same me in every place, I will miss out on many opportunities. With a certain group of people, I’m a Sudanese because I cannot hide that. But with other groups I introduce myself as from another origin otherwise if they knew my Sudanese backgrounds their attitude towards me will change and exclude me — (P1).

To go through a transformation process, even if it’s temporary and artificial, helps to penetrate many local groups to seek integration — (P10).

It’s great I work and mingle with lots of Black people originally from the Caribbean. I feel pretty much like belonging here when I mingle with people like that. People don’t see me as a foreigner — (P2).

Thus, as H Vigh [56] argues,’We act, adjust and attune our strategies and tactics concerning the way we experience and imagine and anticipate the movement and influence of social forces’. Indeed, these migrants were engaged in the process of calculation and recalculation as they sought to integrate into British society.

### Identity fluctuation as a social navigation tool

Writing within the context of young urban men in the West African country of Guinea-Bissau, H Vigh [56] notes that people who live in unstable environments use different tactics and invest a great deal of time in calculating how to use their different positionalities and identities to achieve the most out of their ever-changing environments. Our participants lived in a stable society, but their migrant/refugee identity made their circumstances uncertain; thus, they used different tactics to get the most out of their situations. To this end, some degree of identity concealment appeared in all the interviews—though with varying degrees. The most despised identity was that of a ‘refugee’ in the migrants engaged with identity fluctuation because they had a sense of greater acknowledgement by the host society. The participants honest their opportunity-driven concealment of certain ‘negative’ identities as expressed by some migrants. P9, P5 and P4 explained:

Here, people don’t like refugees. The general view is that refugees are here for the welfare benefits. I don’t mention the term refugee when I speak. No one needs to know about my refugee status. I present myself as everyone else — (P9).

But if I’m isolated because others don’t accept me because they think I’m not making efforts to fit in, that’s not good for my health. I live here for the present. For how many months or years, I do not know; I need to make connections with the locals to survive — (P5).

When you say you are British, they trust you more and think you’re serious about future relationship. But you want other people to connect; that’s important. I suppose you have to adjust — (P4).

Identity dilemmas were pervasive in the migrants’ daily lives because of conflicting but often overlapping and intertwined identities. Many contradictions could be observed between the migrants’ deep cultural and socio-political assumptions and their choices to face reality. An ethical dilemma was about whether to disclose the actual identity or to conceal it. The ideological dilemmas had greater psychosocial ramifications and contradictions because these were profoundly embedded in religious values as well as physical harm. Another dilemma was about self-importance in their national identity, which sharply differed from the transactional mutation into new selves, which were more favourable.

The migrants claimed novel identities in their drive to successfully negotiate the new social space and cultural landscape. However, there were significant barriers to keeping a single identity type.

Telling the truth about your identity’s damaging to your life as that distances you from others — (P11).

I believe that if I didn’t show myself as a British person, I wouldn’t have the job I have. If I say I‘m British in a Sudanese community, my fellow Sudanese will reject me because they might think I am a renegade and I deny my own culture. You’ll not be accepted everywhere with your heavy African accent — (P1).

At the end of the day, we are here. You don’t even know if you’d go back home one day. So, while you are here it’s good to show local people that you are interested in being here and serving this country. So, you’ve got to change — (P6).

P6 and P1 have congruent behaviour, which is reflected through their narratives. P1’s dramatic shift in religious identity was striking. The participant holds the view that Muslim identity is incongruent with British culture. This necessitated his suppression of Islamic value in several social contexts. He adopted a Christian first name because he did not so as not to feel and be labelled as an outsider. P7 (male) and P12 (female), two Muslim migrants, like P1, justified their identity shift:

Deep down I knew if I had shown her (a girl he met) that I was a devout Muslim she wouldn’t go out with me because she likes to have a drink. You know socially a drink is important for Western people — (P7).

People are scared to be with Muslim girl. They don’t understand Islam. They think you’re so different they can’t engage with you. I stopped covering my head and wearing African clothes because I felt that both girls and boys in the school avoided me. I then started to make more friends — (P12).

Similarly, P3 did not feel comfortable talking about her experience of witnessing sexual assault and being subject to humiliation. She strictly avoided talking about her asylum status for fear that she could be asked to explain what happened, triggering the memories she desperately wanted to forget. As she explained:

Only his wife [her uncle’s wife] survived but then it was terrible. She was assaulted several times by soldiers — (P3).

This participant sought the confidence of the locals to narrate her actual story but was confronted with several barriers. Many participants shared these efforts to distance themselves from the ‘negative’ refugee identity. P7 and P13 explained:

I want my new life to be a truly new life — (P7).

The term refugee made me lose lots of good things in life. When you meet a boyfriend the idea in people’s mind is that you’re looking to have British papers. They don’t think about the emotion you have as a human being. Some community members jeer at you when they learn that you’re a refugee. I’ve moved home several times due to that — (P13).

### The subject position espoused by the migrants

As has been shown, our participants wished to assume particular identities, especially those that gave them advantages and acceptability within their new environment. However, as G Valentine and D Sporton [19] argue,’a given identity is not just something that can be claimed by an individual, however; it is also dependent, at least in part, on an individual’s identity being recognised or accepted by a wider community of practice’. Indeed, many of the participants’ identities were both self-constructed and externally imposed by the new country’s socio-political system. The participants adopted different subject positions, which exemplified a variety of identities and attitudes. P1 and P3 saw themselves as forced migrants, which was self-constructed but also forced upon them by the host society. The experience of leaving familiar cultures of the home countries and the pessimism surrounding possible return confined them to the acceptance of their new situation as forced migrants. This subject position was equally attributed to them by host country structures where migrant status was often equated to outsiders and being disadvantaged. P1 and P5 show how the participants constructed this position or how the host society labelled them.

You know I’m a refugee from Sudan. I fled because of the ethnic conflict. And I had to run for my life. I am talking to you as a refugee. As an African, you want to be proud. But being a refugee changes all this —(P1).

I come from Sierra Leone. We tried to bring dad over when we were safe in Britain. I’m a refugee. I don’t think I’d go back to Sierra Leone —(P5).

The migrants did not always select the subject position. Nevertheless, in a number of cases, it was bestowed on them by the social structures that sought to ostracise them. P3 well expressed this external construction of identity:

Knowing I was from there (Sierra Leone) would equate to people knowing that I was a refugee. I didn’t want people to always ask me what happened that I had to flee my country —(P3).

It can be noted that the subject positions adopted were those of ambiguity as they attempt to be simultaneously members of multiples communities, both host and home countries.

It’s not possible for me to forego The Gambia. Social media now let me live Gambian culture better than a few years ago when there was no Facebook, WhatsApp and skype. Even if I feel like I belong in London, I am still Gambian at heart and by blood — (P2).

Some other times I say I’m Sudanese. My fellow Sudanese will reject me. In Sudanese communities, I speak with my real Sudanese accent. I’ve been a Muslim all my life in Sudan. This is part of my culture —(P1).

Denying my Sierra Leonean roots occasionally is just a pretence. It’s a long time now; but my spirit dwells also in Sierra Leone —(P5).

At the same time as being still full-time members of the native communities back home, the participants appropriated subject positions as subject positions aspiring or full members of the host collectivities. The migrants were aware that successful negotiation of the new social and cultural space depended upon demonstrating a commitment to the host society:

On some occasions, I’d say that I’m a British person and apply myself to mirror the local intonation. That way, people embraced me better —(P8).

I started feeling more comfortable here when I gained British citizenship. I then present myself as a British person, I don’t see myself as a liar. British citizenship opened doors —(P15).

The main findings are discussed in the following section, linking key areas of analysis with relevant literature.

## Discussion

The analysis below focuses on showing how the data contribute to answering our central research question: How do individual perceptions of identity affect the lived experiences of forced migrants? D Hack-Polay [57] views self-and external categorisations as the main factors that support the management of identities, which cause contradictions in the behaviours of the subjects who seek new identities. The research examined the way in which the forced migrants steer multiple identities [40], which often signified the discarding or suspension of a certain identity in favour of more beneficial ones was transactional and situated. The findings support the view that identities are not bicultural but far more complex and relational- including differences between migrants, refugees and those settled for longer or shorter time periods [4].

The above quotes not only provide an insight into the conflict and war situations prevalent in the sending countries but also make it conceivable why forced migrants often take desperate measures and dangerous journeys to cross the Mediterranean Sea into Europe. Our participants’ attempts to fit into the new societies are influenced by their past experiences, compounding their apprehensions about exclusion in the host countries. In our study, the migrants’ narratives conveyed their sense and awareness of the ‘anti-asylum-seeker racism’ [58] prevalent in the UK, hence their discomfort as they felt that they were viewed only as (bad) immigrants as opposed to full members of the new communities.

The migrants in our research believed the intermittent or sometimes frequent suspension of the native (or original) selves in the host communities was a purposeful strategy to evade deleterious identities attributed to them. The migrants could then normalise their everyday lives and develop social routines. ‘Refugee’ identity was thus perceived as a liability [28, 39, 41]. Western media have substantially engaged in developing undesirable connotations about the ‘refugee’ identity through much negative coverage. Labelling can be conceptualised as exclusionary to migrants [27, 59]. Alterations in their migrant identity may be geared at escaping socio-cultural exclusion [21, 24]. Setting aside native identities was circumstantial for most migrants, i.e., in the public sphere, because those identities were highly pathologised. The original identities were, therefore, perceived as liabilities and not social capital [60]. The original selves were, however, deployed in private spheres or within migrant enclaves [57].

To safeguard the temporary or espoused identities, the participants refrained from identifying themselves as migrants or foreigners when interacting with the new collectivity. Though the migrants accepted that it was unethical to misrepresent their identities, they felt coerced to do so by the social system and institutional structures. They did not doubt the legitimacy of espousing new and circumstantial identities as this is a matter of social, psychological and economic survival. This afforded them a degree of dignity in their new communities. The participants perceived ‘migrant identity’ as counter-productive [19, 27, 61]. British-ness, in contrast, appeared to be a desirable identity in the participants’ eyes. The positions that the migrants took differed based on their perceived social value [34]. Within migrant circles, the participants deployed their actual migrant identity. However, when interacting socially or economically with the host communities, British identity was favoured temporarily by many migrants for the purpose of successful negotiation of the host environment and sense of belongingness [39, 62].

The forced migrant participants largely rejected the ‘refugee’ identity when interacting with the new community due to the negative connotation attached to it. Negative identity could lead them to experience undignified treatment in the new social context, resulting from ‘othering’ [63]. The perceived stigma associated with ‘refugee’ identity has been widely studied in the field of help-seeking behaviour, particularly in the context of the barriers to accessing mental health services in the host country, due to cultural beliefs or the fear of mistreatment [64, 65]. Several current global campaigns and policies for the benefit of refugees often call for more dignity for this group. Such campaigns include “Dignity not Destitution” [66] and “Respect for All” [67]. Drawing on our participants’ narratives, this could be interpreted as a positive wave of interventions, shaped by present identity narratives, given that the anxiety of the profanation of their dignity impacts significantly on the forced migrants’ appropriation and discarding of identities.

In total, identity fluctuation became a significant social navigation strategy for our participants. However, the exaltation of espoused identities (particularly citizenship) meant accepting the utilitarian role of adopted selves [68]; even pathologised identities in contemporary British society, e.g. foreigners, refugees, immigrants, deprived, and so forth, could become useful depending on context. All the participants in the study are from Africa, and several of the example transcripts discuss their race. Race, thus, appears to be a factor strengthening the participants’ rejection of the pathologised ‘refugee’ identity, whose perceived adverse effect could be compounded in an already racialised British society. Therefore, to a large extent, the migrants were led to use identity fluctuation, especially new migrants’ rights became restricted in the UK and much of the European Union. Often, gaining citizenship status assisted the change of identity socially and psychologically. For example, the forced migrants narrated how they laboured hard to acquire minority British accents to disguise their foreignness. Indeed, C Antaki, S Condor and M Levine [69] argue that identity can be situated in conversational interaction, whereby speakers can draw on fluctuating identities in order to invoke both group distinctiveness and similarity, arguably as a mode of social navigation.

Further, the longer our participants lived in the new communities, the more the forced migrants moved towards hybridity of identity. This correlates with the bicultural perspective on identity presented by C Ward, C Ng Tseung-Wong, A Szabo, T Qumseya and U Bhowon [24]. They found that hybrid and alternating identities served as valuable tools in the struggle to find a place in a multicultural context [24]. Identity fluctuation happened in much of the migrant social realities, namely behaviours, language, religion, social interactions, values and drinking and eating habits, and so forth. In several cases, migrants engaged in identity ‘change’ because they saw it as an imperative action for social integration. Some scholars [68, 70–72] contend that, in different cultural contexts, people generally show a penchant for identities associated with the dominant culture. To a large extent, social media (Facebook, Instagram, WhatsApp, etc.) helped the participant negotiate different identities [73]. Nevertheless, they remained close to their native ones as they maintained ties ‘back home’ through live participation in festivals, meetings and similar left-behind cultures.

The participants saw language as an important identity factor, maintaining that common phrases, adages and accents, must fit or support the re-engineered self in order to penetrate the new social order. For R Mitchell, F Myles and E Marsden [74], there is a gap between first-generation and subsequent generations of migrants in terms of host language competence as the second language develops considerably to the detriment of the migrant’s native langue (especially in second generations). To substantiate this, D Bhugra and MA Becker [28] claim that the second generation of forced migrant offspring realised that cultural transformation was imperative for survival. This enabled them to develop greater English language competence than their first-generation parents.

Religion could aid the socialisation process [37] and represent a remedy for social exclusion and isolation; the host cultural paradigm exercises some dominance over time. Some authors [57, 71] found that the migrant population—and minorities more broadly—lean towards the dominant culture over time. The participants in our study largely attempted to retain the religious identity they arrived in exile with. However, close ties with the original religious identity also diminished in the long term. Like P1 and P14, many participants perceived having ‘social times’ with locals as forced compliance with the host country’s cultural patterns, using these as strategies to create opportunities [25, 61]. This supports TL Pittinsky, M Shih and N Ambady [75], concerning the notion that identities are situated. The erosion of original cultural norms is explained by MM Gordon [76] in what the author termed the Anglo-conformity assimilation model. This model casts light on the coercion that migrants face to conform to the locality as a condition for social, economic and political inclusion (see also [32, 77]). Non-compliant migrants, however, will experience more difficulties in ‘gaining recognition and surviving’ [16, 40]. Social integration necessitates a systematic appraisal of host realities by the migrants in order to establish where to position themselves [78–80].

The way in which the migrants experienced identity oscillation was clearly inherent to the migrant integration process. In this process, identities that enjoyed a positive perception in the eyes of the migrants were espoused to facilitate the migrants’ navigation of the new social context, leading to the expectation of social promotion [40, 57].

Narratives provided by the migrants aided the explication of the range of identities and socio-cultural routing strategies. Our research extends the literature regarding responses that migrants develop that may cause conflict between the migrant groups and locals. The findings highlight the way in which migrants in a given society articulate varied approaches geared at testing the beliefs the hosts hold about newcomers. Such an analysis of epitomises is the complexity of the identity issue [81]. This equally exemplifies the extent to which new identities develop organically, are context-dependent and evolutionary. These are also affected by the context leading to the forced migrants leaving their countries, typically socio-political conflict. Our study participants were from nine countries that have experienced varying degrees of conflict. Within the scope of this paper and for the purpose of brevity, the details of the nature of these conflicts have been omitted. Instead, we have elected to provide context for those that the participants mentioned explicitly as part of their reasons for leaving their country to explain how these pre-exile circumstances shaped their identities in the host countries.

## Conclusion

The investigation started with the overarching research question about how individual perceptions of identity can affect the lived experiences of forced migrants. The findings show that forced migrants navigate their new cultural and institutional settings by articulating identities that are unconsciously or consciously espoused and expressed. Fluctuating identities are necessitated by constraints of the host environment that may tend to use the migrant status as a basis for exclusion of the newcomers. This finding elucidates our overarching research question, portraying the forced migrants’ perception of refugee identity as a liability. This demonstrates that identities, in many respects, derive from conscious construction. Whether provisional or permanent, setting aside native identities among migrants socially and psychologically situated [21, 24]. The construction of normality (or at least a new normal) constitutes the main basis for the migrants’ repudiation of pathologised migrant identities or the appropriation of desirable identities as reminiscent survival tactics for sense-making in readiness to fill novel social and economic roles.

The formation of the new identity is a process of reality construction that goes on until the migrants develop effective social navigation of the host terrain. As they become settled, their original selves are re-evaluated in the light of novel constraints, temporarily suspending old identities and appropriating more contextually valid identities (see [35]). In this perspective, there develops an inferiority-superiority belief that endorses the supposed superior or desirable identity that is significantly more opportunity-driven. Identity fluctuation was found to be contingent upon the context, thus rejecting the argument of fixed identities [34]. This indicates the plausibility of the argument that the appropriation of sporadic selves is largely situated in time and space. Our research represents a novel contribution to the literature on migrant identity practice. A paucity of scholarship connects the notion of the stigma associated with forced migrant status and the extent to which forced migrants appraise their reception in exile as undignified. This study remedies some weaknesses in the literature; ascertaining identity fluctuation may not be unidirectional, indicating that shifting towards positive identity is not the only position people take. Identity fluctuation is geared at the opportunity from economic, social and economic standpoints. Examining migrant identities from an interpretivist perspective allows researchers to apprehend the diverse facets of identity as far as migrants are concerned. This endeavour requires multiple research frameworks to elucidate migrants’ complex identity shift exercise or choice of identity (which we termed identity fluctuation in the study). Additionally, our inquiry was not explicitly intended to examine how migrants’ race, gender and identity could interact—adding another limitation that should be acknowledged here. Future research could explore this intersection, drawing on large samples that would allow multi-group analyses and employing quantitative (or mixed-) methods that would offer the support of inferential statistics to judge the variance in the forced migrants’ perceptions of identity as a result of the interaction with relevant moderating variables, e.g. gender, generational cohort, education, social media, extent of prior trauma, and many more.

## Data Availability

All data generated or analysed during this study are included in this published article.

## Abbreviations

UNHCR: United Nations High Commissioner for Refugees
P: Participant
